# Integrating transcriptomics and proteomics to show that tanshinone IIA suppresses cell growth by blocking glucose metabolism in gastric cancer cells

**DOI:** 10.1186/s12864-015-1230-0

**Published:** 2015-02-05

**Authors:** Li-Ling Lin, Chieh-Ren Hsia, Chia-Lang Hsu, Hsuan-Cheng Huang, Hsueh-Fen Juan

**Affiliations:** Department of Life Science, National Taiwan University, No. 1, Sec. 4, Roosevelt Road, Taipei, 106 Taiwan; Institute of Biomedical Informatics and Center for Systems and Synthetic Biology, National Yang-Ming University, No.155, Sec.2, Linong Street, Taipei, 112 Taiwan; Institute of Molecular and Cellular Biology, National Taiwan University, No. 1, Sec. 4, Roosevelt Road, Taipei, 106 Taiwan; Graduate Institute of Biomedical Electronics and Bioinformatics, National Taiwan University, No. 1, Sec. 4, Roosevelt Road, Taipei, 106 Taiwan

**Keywords:** Tanshinone IIA, Gastric cancer, Glycolysis, Isobaric tags for relative and absolute quantification, Next generation sequencing

## Abstract

**Background:**

Tanshinone IIA (TIIA) is a diterpene quinone extracted from the plant Danshen (*Salvia miltiorrhiza*) used in traditional Chinese herbal medicine. It has been reported to have anti-tumor potential against several kinds of cancer, including gastric cancer. In most solid tumors, a metabolic switch to glucose is a hallmark of cancer cells, which do this to provide nutrients for cell proliferation. However, the mechanism associated with glucose metabolism by which TIIA acts on gastric cancer cells remains to be elucidated.

**Results:**

We found that TIIA treatment is able to significantly inhibit cell growth and the proliferation of gastric cancer in a dose-dependent manner. Using next-generation sequencing-based RNA-seq transcriptomics and quantitative proteomics-isobaric tags for relative and absolute quantification (iTRAQ), we characterized the mechanism of TIIA regulation in gastric cancer cell line AGS. In total, 16,603 unique transcripts and 102 proteins were identified. After enrichment analysis, we found that TIIA regulated genes are involved in carbohydrate metabolism, the cell cycle, apoptosis, DNA damage and cytoskeleton reorganization. Our proteomics data revealed the downregulation of intracellular ATP levels, glucose-6-phosphate isomerase and L-lactate dehydrogenase B chains by TIIA, which might work with disorders of glucose metabolism and extracellular lactate levels to suppress cell proliferation. The up-regulation of p53 and down-regulation of AKT was shown in TIIA- treated cells, which indicates the transformation of oncogenes. Severe DNA damage, cell cycle arrest at the G_2_/M transition and apoptosis with cytoskeleton reorganization were detected in TIIA-treated gastric cancer cells.

**Conclusions:**

Combining transcriptomics and proteomics results, we propose that TIIA treatment could lead cell stresses, including nutrient deficiency and DNA damage, by inhibiting the glucose metabolism of cancer cells. This study provides an insight into how the TIIA regulatory metabolism in gastric cancer cells suppresses cell growth, and may help improve the development of cancer therapy.

**Electronic supplementary material:**

The online version of this article (doi:10.1186/s12864-015-1230-0) contains supplementary material, which is available to authorized users.

## Background

Gastric cancer is one of the most notorious cancers worldwide. It is the fourth most frequently occurring cancer, and the second most common cause of death for both sexes among all cancers, claiming over 736,000 lives worldwide in 2008 [1]. However, anti-cancer drug development of gastric cancer today remains slow and costly, and drug-resistance remains a potential obstacle [2].

Tanshinone IIA (TIIA) is a diterpene quinone extracted from the plant Danshen (*Salvia miltiorrhiza*), which is used in traditional Chinese medicine [3]. The first usage of Danshen extractions as herbal medicine can be traced back to more than 1800 years ago [4]. Its applications include prevention of cardiac diseases [5], protection of the nervous system [6] and hepatocytes [7], and inhibition of osteoporosis [8]. Drug repositioning is considered to be a promising and valuable method for the reduction of the side effects and cost of anti-cancer drug research and development. The functions of many chemicals extracted from Danshen are therefore explored for their anti-cancer potential, and TIIA is the most abundant and structurally representative of these [9]. TIIA has been recently reported to have anti-cancer potential against several cancers including breast cancer [10], prostate cancer [11], colorectal cancer [12], lung cancer [13], liver cancer [14], leukemia [15], and gastric cancer [16]. However, the regulatory mechanism of TIIA in gastric cancer cells remains unclear.

Metabolic transformation accompanying nutritional imbalance is one of the leading causes of cancer progression. Glucose is a primary source for the pentose phosphate pathway, which makes RNA and DNA. Glycolytic intermediates can be used to assist lipid biosynthesis to produce ATP and non-essential amino acids, such as alanine, for growth [17]. Proliferating cells are commonly maintained by enhancing aerobic glycolysis, also called the Warburg effect, which causes lactate accumulation and contributes to the development of malignancies. In oncogenic pathways, phosphatidylinositide 3-kinases (PI3K)/protein kinase B (AKT) has been shown to enhance glycolysis, while the tumor suppressor p53 inhibits it, suggesting a glycolytic switch intrinsically associated with oncogenic transformation [18]. Through the inference of oncogene expression and glucose metabolism, the supply of nutrients to cancer cells could be blocked, which would stunt their proliferative potential. This may be an important finding for drug discovery.

High-throughput data can be used to provide a comprehensive inventory of all the biological processes of cells, display their complexity, and increase data accuracy. An accurate picture of the differential expression of experimental samples is important for defining precise targets and networks. Here we use two types of high-throughput data to uncover the regulatory mechanism of TIIA in gastric cancer cells: transcription levels from next-generation sequencing (NGS) data, and isobaric tags for relative and absolute quantification (iTRAQ)-based quantitative proteomics analysis. NGS is one of newer transcriptome sequencing approaches and can perform high-throughput sequencing by generating thousands or millions of sequences in parallel [19]. It has better sensitivity and lower background noise than microarray analysis, and more researchers are using it to investigate the mechanisms of anti-cancer drugs [19,20]. iTRAQ is a promising new technique for quantitative proteomics which can quantify proteins from different sources using their different tags [21]. Because of its sensitivity in labeling peptides, it is considered a more powerful quantitative proteomic technique than others, including the two frequently used quantitative proteomics techniques 2D-DIGE (difference gel electrophoresis) and cICAT (cleavable isotope-coded affinity tags) [22,23].

In this study, we identified a total of 16,603 unique transcripts and 102 TIIA-regulated proteins that are involved in glucose metabolic process. We further investigated and found that TIIA blocks glycolysis and gluconeogenesis in cancer cells by altering protein expression, and causes DNA damage, cell cycle arrest, cytoskeleton reorganization and apoptosis.

## Results

### TIIA reduces the growth rate and suppresses proliferation of gastric cancer AGS cells

**Figure 1:**
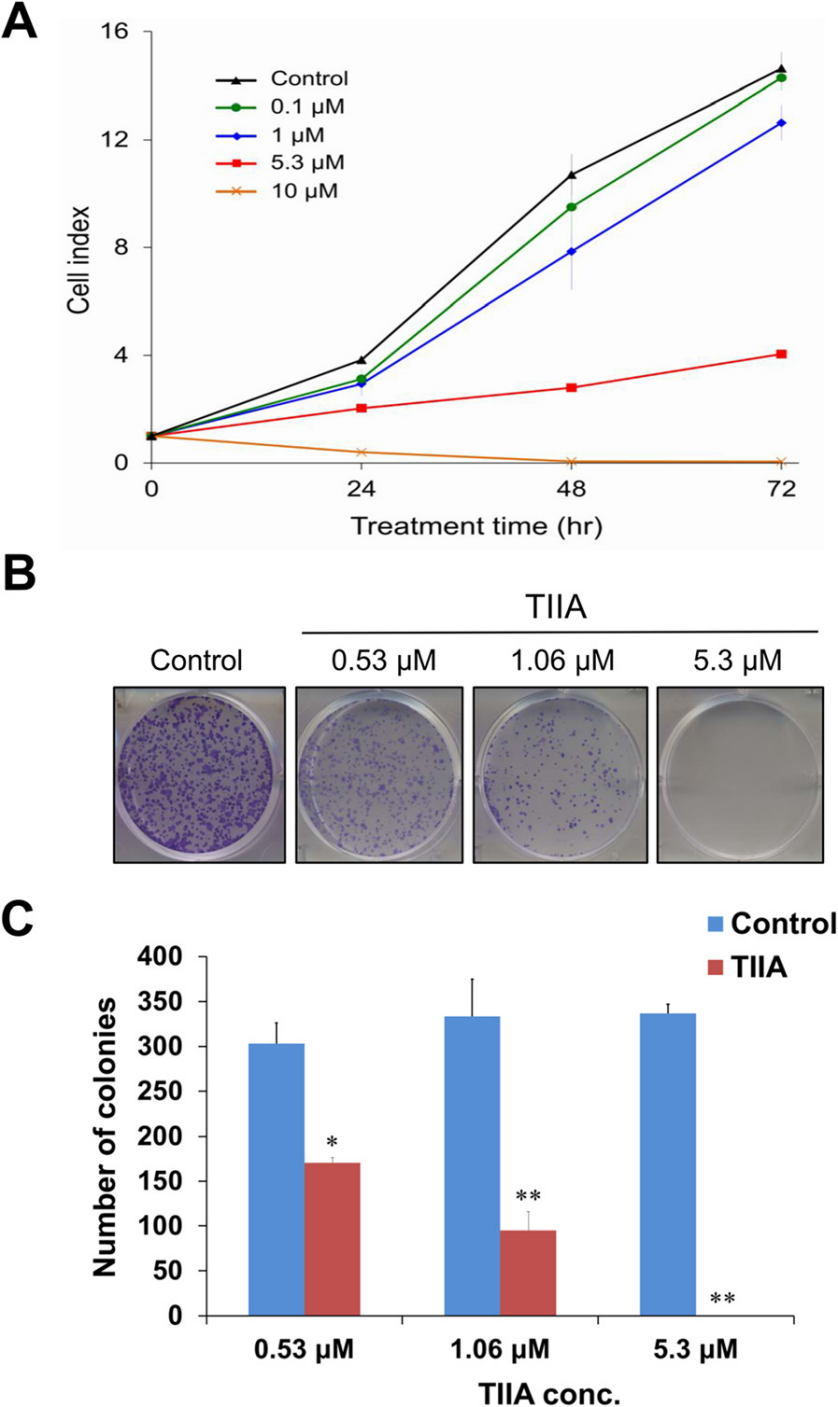
**Effects of TIIA on cell growth and proliferation of AGS cells. (A)** Growth curves show a dosage-dependent pattern of growth inhibition after TIIA treatment for 72 hr. All TIIA treatment conditions had significant effects on AGS cell growth (*p* < 0.0001, Wilcoxon Signed-Rank Test). Cell indexes of AGS cells were measured by an RTCA DP® system, expressed as mean ± SD of three replications. **(B)** Colonies of AGS cells were stained by crystal violet (purple-colored dots) after TIIA treatment. Control conditions exhibit many colonies, while IC_50_ TIIA conditions have produced almost no colonies. **(C)** TIIA significantly reduces the number of AGS colonies (*p < 0.05, **p < 0.01, Student’s *t*-Test) in a dosage-dependent manner. Histogram values are expressed as mean ± SD from three replications.

To determine whether TIIA can affect gastric cancer cell survival, we treated AGS cells with different concentrations to detect real-time cell growth rates by an RTCA (Real-Time Cell Analyzer; xCELLigence™; Roche Applied Science & ACEA Biosciences) system. The IC_50_ of TIIA at 48 hr was calculated to be 5.3 μM. Figure 1A shows that 0.1 μM TIIA was only slightly effective, whereas 10 μM TIIA killed almost all cells. Each curve is significantly different from the other (*p* < 0.0001, Wilcoxon Signed-Rank Test). Our results demonstrate that the cell growth rate was significantly decreased under TIIA treatment conditions compared with controls, suggesting that AGS cell growth was significantly inhibited by TIIA in a dose-dependent manner.

To explore whether TIIA could affect gastric cancer cell proliferation rates, we treated AGS cells at different concentrations of TIIA and counted the number of cell colonies stained with crystal violet. The results show that 0.53 μM (1/10 IC_50_) TIIA reduces colonies to about 60%, whereas 5.3 μM (IC_50_) TIIA results in almost no colonies. It indicates that the proliferation rate of AGS cells was significantly suppressed by TIIA in a dose-dependent manner (*p < 0.05, **p < 0.01, Student’s *t*-test) (Figure 1B and C).

### Functional annotation enrichment of TIIA -regulated genes

**Figure 2:**
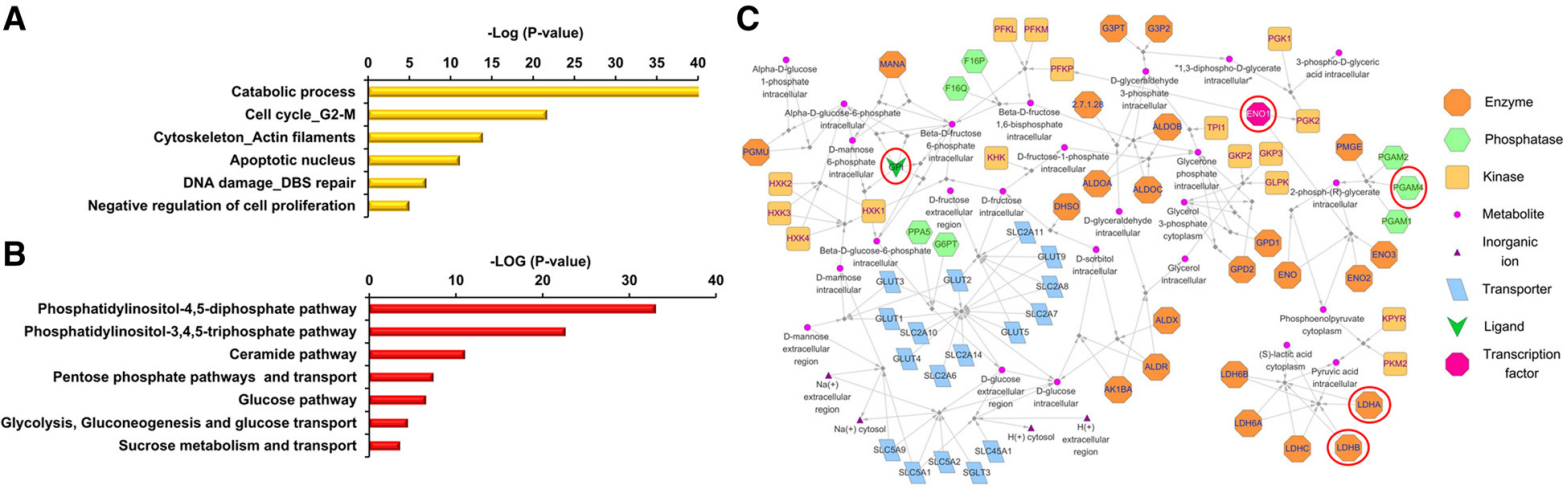
**Enrichment analyses of the biological processes of TIIA-regulated genes.** Analysis of TIIA-regulated genes with MetaCore software enriched the biological processes. **(A)** Classification of all identified genes collected from RNA-seq into process networks. **(B)** Classification of all identified genes collected from RNA-seq into metabolic networks. **(C)** The “*Glycolysis, gluconeogenesis and glucose transport*” network built from **(B)**. Enzymes expressed at RNA-seq and iTRAQ are circled in red for clarity. Significance calculated by MetaCore was plotted as the negative log of the *p* value.

To uncover the TIIA regulatory mechanism in AGS cells, we performed RNA-seq analysis to profile the transcriptomes of gastric cancer cells when treated with dimethyl sulfoxide (DMSO) (control) or with TIIA. In order to characterize the gene expression profile to response of biological functions, our initial analysis performed functional enrichment of all identified genes onto MetaCore pathway analysis. Major bio-functional networks were significantly enriched and showed in Figure 2A. Of six bio-functional networks, “*Catabolic process*” is top-ranked. Previous studies have shown that dysregulation of metabolism is an important indicator for tumorgenesis [17]. Thus, we forced on metabolic networks to enrich all identified genes by MetaCore. As indicated in Figure 2B, a large number of glucose networks were affected, including *Phosphatidylinositol-4,5-diphosphate pathway*, *Phosphatidylinositol-3,4,5-triphosphate pathway, Ceramide pathway, Pentose phosphate pathways and transport, Glucose pathway, Glycolysis, Gluconeogenesis and glucose transport,* and *Sucrose metabolism and transport*. It has been known that PI3K, an key enzyme of phosphatidylinositol-4,5-diphosphate and phosphatidylinositol-3,4,5-triphosphate pathways, increases glycolysis in cancer cells [18]. Based on these results, we propose that TIIA-regulated pathways involving in the enriched *Glycolysis, Gluconeogenesis and glucose transport* network (Figure 2C).

Moreover, a total of 2,761 differentially expressed genes (DEGs) were considered that have different expression between control and TIIA treatment samples (FDR ≤ 0.001 and |log_2_(fold-change)| ≥ 1) (Additional files 1 and 2). Among these, 1,010 genes were up-regulated and 1,751 genes were down-regulated by TIIA. MetaCore networks analysis of DEGs reveals significantly enriched biological processes that frequently occur in gastric cancer cells with TIIA treatment. As shown in Table 1 and Additional file 3, major bio-functional networks were in correspondence with the enrichment analysis of integrative transcriptome (Figure 2A), such as *Cell cycle_G2-M*, *DNA damage_DBS repair*, *Apoptotic nucleus*, *Cytoskeleton_Intermediate filaments*.

**Table 1:** **Enrichment analysis of RNA-seq-identified DEGs by MetaCore software**

**Function description (number of genes)**	***P*** **-value**	**Differentially expressed genes (DEGs)**
Cell cycle_G2-M (75)	9.611E-13	Cyclin G, DNMT3B, Ubiquitin, TOP2 beta, ANAPC4, Condensin, UBC, Brca1, Cyclin B, PDGF-B, Cyclin B2, Brca2, Histone H3, HUS1, Miz-1, CDC25C, PTCH1, TRF1, Cyclin A2, BLM, CAP-G, PLK4 (STK18), FANCD2, CAP-G/G2, 14-3-3 sigma, EGFR, GTSE1, ATM, MAD2a, FOXM1, Lamin B1, GADD45 beta, 14-3-3, BUBR1, TOP2 alpha, CAP-H/H2, PDGF-A, LATS2, Lamin B, ETS2, GADD45 alpha, CAP-C, Cyclin A, CNAP1, Rad50, Cyclin G2, DNMT1, Skp2/TrCP/FBXW, CAP-D2/D3, KNSL1, Aurora-B, CDC25A, SKP2, DNMT3A, TOP2, PDGF-R-alpha, Dynamin, p21, BUB1, BRRN1, PDGF receptor, ATR, Cyclin B1, Emi1, c-Myc, Claspin, Aurora-A, CDC25, ATRIP, PKA-cat (cAMP-dependent), CAP-E, Histone H1, CDK1 (p34), CDK7, CDC27
DNA damage_DBS repair (44)	1.260E-08	Ubiquitin, TOP2 beta, Tip60, Sirtuin, MRE11, Brca1, BRIP1, Brca2, MeCP2, Histone H3, MRN complex, C1D, HMGB1, BLM, FANCD2, RAD54B, RAD54L, SMC6, ATM, DNA ligase IV, DNA-PK, NEK1, TOP2 alpha, FANCA, Histone H2AX, SMC5, PP2A regulatory, Rad50, ChAF1 subunit B, XRCC2, FANCL, TOP2, Sirtuin6, ATR, PSF, DNA polymerase eta, FANCM, PIR51, Nibrin, HMG1,2, WRN, ATRIP, Histone H4, p53BP1
Apoptosis_Apoptotic nucleus (49)	3.077E-06	Tip60, NRIF3, NF-kB2 (p100), DNA polymerase kappa, RelA (p65 NF-kB subunit), Brca1, PARP-1, Tubulin alpha, TIA-1, Separase, Histone H3, ROCK2, Caspase-6, BLM, FANCD2, iASPP, C/EBPbeta, Granzyme B, IEX1, ATM, Histone H2B, IRF5, DOCK1, DNA ligase IV, FOXO3A, DNA-PK, c-Myb, c-Jun, Protein p8, Lamin B1, GADD45 beta, Clusterin, DFF40 (CAD), PHAP1 (pp32), Lamin B, UBE1C, GADD45 alpha, Axin1, NF-kB, p21, ATR, P53DINP1a, Bcl-6, Bard1, APP-BP1, Nibrin, XPD, Perforin, Histone H1
Cytoskeleton_Intermediate filaments (30)	4.660E-06	Plectin 1, Tubulin beta, Keratin 17, BPAG2, Desmin, PPL(periplakin), ROCK, Tubulin alpha, SYNE2, ROCK2, Keratin 18, Keratin 19, Keratin 8/18, Lamin B1, 14-3-3, Lamin B, Keratin 8, Nestin, Keratin 6A, BPAG1, TMPOA, TMPOB, Kinesin light chain, Plakophilin 2, Actin, Fimbrin, Peripherin, CDK1 (p34), Tubulin (in microtubules), Nesprin 1

### Proteomic expression profiling of TIIA-treated gastric cells

To further elucidate cellular mechanism and molecular function, we performed iTRAQ-based proteomics analysis to assess the protein expression profiles in AGS cells with TIIA treatment. Equal amounts of peptides collected from control samples or TIIA-treated samples were labeled with iTRAQ reagents, respectively, and used for replication (control samples labeled by 114 or 115; TIIA-treated samples labeled by 116 or 117). All labeled peptides were mixed and analyzed by liquid chromatography coupled with tandem mass spectrometry (LC-MS/MS). A total of 102 differentially expressed proteins were identified with a false discovery rate (FDR) of 3.94%; annotated MS/MS spectra are collected in Table 2 and Additional file 4. There were 100% of identified peptides that were labeled with iTRAQ reagents. The intensity levels of iTRAQ signals distribution plots show that positive correlation among our treatments (Additional file 5), suggesting that labeled samples with a high reproducibility were present in this study.

**Table 2:** **List of iTRAQ-identified proteins regulated by TIIA**

**No.**	**Biological function**	**Accession**	**Protein name**	**Gene symbol**	**Protein score**	**No. of quantified peptides**	**Coverage (%)**	**iTRAQ ratio**	**RNA-Seq ratio**
1	Glycolysis	P11166	Solute carrier family 2, facilitated glucose transporter member 1	SLC2A1	72	2	3	1.273	1.573
2	Glycolysis	Q8N0Y7	Probable phosphoglycerate mutase 4	PGAM4	40	1	5.5	1.057	0.309
3	Glycolysis	P06733	Alpha-enolase	ENO1	720	15	46.8	0.947	0.754
4	Glycolysis	P00338	L-lactate dehydrogenase A chain	LDHA	283	6	29.2	0.901	0.352
5	Glycolysis	P04406	Glyceraldehyde-3-phosphate dehydrogenase	GAPDH	813	14	55.2	0.823	0.934
6	Glycolysis	P07195	L-lactate dehydrogenase B chain	LDHB	274	9	29	0.736	0.553
7	Glycolysis	O60701	UDP-glucose 6-dehydrogenase	UGDH	49	1	4.3	1.001	1.938
8	Glycolysis	P06744	Glucose-6-phosphate isomerase	GPI	83	1	7.3	0.525	0.824
9	ATP metabolic process	P55072	Transitional endoplasmic reticulum ATPase	VCP	129	4	7.3	0.888	0.661
10	ATP metabolic process	P04075	Fructose-bisphosphate aldolase A	ALDOA	196	5	30.5	0.804	1.284
11	Protein folding	P14625	Endoplasmin	HSP90B1	67	1	8.7	1.685	0.738
12	Protein folding	P60709	Actin, cytoplasmic 1	ACTB	1161	30	47.2	1.129	0.795
13	Protein folding	P62937	Peptidyl-prolyl cis-trans isomerase A	PPIA	242	6	42.4	1.050	0.640
14	Protein folding	P23284	Peptidyl-prolyl cis-trans isomerase B	PPIB	69	2	11.6	1.084	0.988
15	Protein folding	P10809	60 kDa heat shock protein, mitochondrial	HSPD1	522	7	21.6	1.011	0.724
16	Protein folding	P07900	Heat shock protein HSP 90-alpha	HSP90AA1	614	7	27.7	0.991	0.572
17	Protein folding	P38646	Stress-70 protein, mitochondrial	HSPA9	71	1	4.1	1.037	0.946
18	Protein folding	P11142	Heat shock cognate 71 kDa protein	HSPA8	728	18	43.7	0.898	0.804
19	Protein folding	P50991	T-complex protein 1 subunit delta	CCT4	55	1	4.5	0.890	0.883
20	Protein folding	P49368	T-complex protein 1 subunit gamma	CCT3	56	1	4.8	1.119	0.876
21	Protein folding	P50454	Serpin H1	SERPINH1	43	1	7.9	0.870	0.568
22	Protein folding	Q99832	T-complex protein 1 subunit eta	CCT7	38	1	7.7	0.896	0.812
23	Protein folding	Q15084	Protein disulfide-isomerase A6	PDIA6	44	1	3.4	0.910	0.876
24	Protein folding	P08238	Heat shock protein HSP 90-beta	HSP90AB1	549	5	32.6	0.783	0.738
25	DNA damage response	Q96QE3	ATPase family AAA domain-containing protein	ATAD5	44	1	3.4	2.004	0.307
26	DNA damage response	P49720	Proteasome subunit beta type-3	PSMB3	105	1	8.8	1.799	0.765
27	DNA damage response	P62979	Ubiquitin-40S ribosomal protein S27a	RS27A	46	1	21.2	1.115	NA
28	DNA damage response	Q96QV6	Histone H2A type 1-A	HIST1H2AA	205	1	40.5	1.006	NA
29	Cell proliferation	Q06830	Peroxiredoxin-1	PRDX1	114	3	31.2	1.098	0.987
30	Cell proliferation	P22392	Nucleoside diphosphate kinase B	NME2	142	5	47.4	0.924	NA
31	Cell cycle	P85299	Proline-rich protein 5	SMR3A	39	1	1.8	1.048	NA
32	G2/M transition of mitotic cell cycle	P07437	Tubulin beta chain	TUBB	660	5	31.8	0.927	0.508
33	Apoptotic process	P02545	Prelamin-A/C	LMNA	56	2	5.9	1.585	1.457
34	Apoptotic process	P23528	Cofilin-1	CFL1	101	1	17.5	1.503	0.735
35	Apoptotic process	P05783	Keratin, type I cytoskeletal 18	KRT18	350	8	36	1.327	2.884
36	Apoptotic process	P04264	Keratin, type II cytoskeletal 1	KRT1	1877	38	10.7	1.088	NA
37	Apoptotic process	O43707	Alpha-actinin-4	ACTN4	324	10	14.3	1.054	1.560
38	Apoptotic process	P61978	Heterogeneous nuclear ribonucleoprotein K	HNRNPK	221	5	16.6	0.938	0.760
39	Apoptotic process	P30101	Protein disulfide-isomerase A3	PDIA3	40	1	9.7	0.702	0.889
40	Apoptotic process	P11021	78 kDa glucose-regulated protein	HSPA5	191	3	22.3	0.633	1.859
41	Angiogenesis	P19338	Nucleolin	NCL	242	6	20.4	1.242	0.504
42	Angiogenesis	P07355	Annexin A2	ANXA2	412	14	51.9	1.217	1.692
43	Cytoskeleton organization	P15311	Ezrin	EZR	129	4	17.6	1.377	2.328
44	Cytoskeleton organization	P08727	Keratin, type I cytoskeletal 19	KRT19	404	3	39.3	1.329	2.486
45	Cytoskeleton organization	P05787	Keratin, type II cytoskeletal 8	KRT8	813	19	52	1.317	2.486
46	Cytoskeleton organization	P07737	Profilin-1	PFN1	281	5	34.3	0.923	0.696
47	Actin crosslink formation	P21333	Filamin-A	FLNA	64	1	2.2	1.055	1.644
48	Microtubule cytoskeleton organization	Q9BQE3	Tubulin alpha-1C chain	TUBA1C	1208	23	45.7	0.869	0.717
49	DNA repair	P06748	Nucleophosmin	NPM1	145	3	21.1	0.973	0.785
50	DNA ligation	P12956	X-ray repair cross-complementing protein 6	XRCC6	76	2	4.4	0.841	0.746
51	DNA repair	Q13263	Transcription intermediary factor 1-beta	TRIM28	68	2	4.8	1.021	0.841
52	DNA replication	P55209	Nucleosome assembly protein 1-like 1	NAP1L1	61	1	7.4	0.913	0.895
53	DNA replication	Q01105	Protein SET	SET	77	1	3.4	0.826	0.613
54	Nucleosome assembly	P04908	Histone H2A type 1-C	HIST1H2AB	175	2	37.7	1.166	NA
55	Nucleosome assembly	Q99879	Histone H2B type 1-M	HIST1H2BM	355	9	55.6	1.073	NA
56	Nucleosome assembly	Q16695	Histone H3.1 t	HIST3H3	95	5	35.3	0.991	NA
57	Nucleosome assembly	P62805	Histone H4	HIST2H4A	406	14	51.5	0.991	NA
58	Translational elongation	P46783	40S ribosomal protein S10	RPS10	82	2	5.5	1.862	1.066
59	Translational elongation	P46777	60S ribosomal protein L5	RPL5	36	1	4.7	1.389	1.106
60	Translational elongation	P08865	40S ribosomal protein SA	RPSA	61	1	14.6	1.243	1.296
61	Translational elongation	P62277	40S ribosomal protein S13	RPS13	95	2	23.2	1.014	1.479
62	Translational elongation	P61313	60S ribosomal protein L15	RPL15	53	1	5.9	0.983	1.129
63	Translational elongation	P32969	60S ribosomal protein L9	RPL9	78	1	18.8	0.838	1.508
64	Translational elongation	P23396	40S ribosomal protein S3	RPS3	69	3	23.9	0.845	1.240
65	Translational elongation	P13639	Elongation factor 2	EEF2	451	7	24.1	0.796	2.148
66	Translational elongation	P62249	40S ribosomal protein S16	RPS16	65	1	25.3	1.042	1.397
67	Translational elongation	P50914	60S ribosomal protein L14	RPL14	80	1	25.6	0.744	1.316
68	Translational elongation	P39019	40S ribosomal protein S19	RPS19	76	2	31	0.702	1.710
69	Translational elongation	Q02543	60S ribosomal protein L18a	RPL18A	50	1	5.1	0.709	1.369
70	Translational elongation	P46778	60S ribosomal protein L21	RPL21	58	1	9.4	0.609	1.377
71	Translational elongation	P18124	60S ribosomal protein L7	RPL7	58	1	11.3	0.538	1.304
72	Translational elongation	P15880	40S ribosomal protein S2	RPS2	38	1	11.3	0.333	1.370
73	RNA metabolic process	P55010	Eukaryotic translation initiation factor 5	EIF5	51	1	3.5	2.675	1.248
74	RNA metabolic process	P07910	Heterogeneous nuclear ribonucleoproteins C1/C2	HNRNPC	158	2	20.3	0.917	0.682
75	RNA metabolic process	O14979	Heterogeneous nuclear ribonucleoprotein D-like	HNRNPDL	63	2	6.4	0.810	NA
76	RNA metabolic process	Q32P51	Heterogeneous nuclear ribonucleoprotein A1-like 2	HNRNPA1L2	250	7	29.4	0.764	1.117
77	RNA metabolic process	P60842	Eukaryotic initiation factor 4A-I	EIF4A1	115	2	16.3	0.725	0.595
78	RNA metabolic process	P52597	Heterogeneous nuclear ribonucleoprotein F	HNRNPF	116	2	9.6	0.647	0.699
79	Epidermis development	P02533	Keratin, type I cytoskeletal 14	KRT14	266	1	21.4	2.455	0.667
80	Epidermis development	P35527	Keratin, type I cytoskeletal 9	KRT9	506	16	26.6	1.072	NA
81	Ectoderm development	P04259	Keratin, type II cytoskeletal 6B	KRT6B	432	1	23	1.225	22.537
82	Sulfur amino acid metabolic process	P23526	Adenosylhomocysteinase	AHCY	43	1	9.7	1.798	0.967
83	Epithelial to mesenchymal transition	Q99729	Heterogeneous nuclear ribonucleoprotein A/B	HNRNPAB	62	1	2.4	1.640	0.543
84	Energy reserve metabolic process	P05141	ADP/ATP translocase 2	SLC25A5	58	1	5.7	1.412	1.234
85	Embryo development	P60174	Triosephosphate isomerase	TPI1	63	1	4.5	1.362	0.636
86	NADH metabolic process	P40926	Malate dehydrogenase, mitochondrial	MDH2	46	1	3.3	1.262	1.022
87	GTP catabolic process	P68371	Tubulin beta-4B chain	TUBB4B	490	2	31	1.216	0.572
88	Cellular membrane organization	P63104	14-3-3 protein zeta/delta	YWHAZ	224	2	34.3	1.208	1.016
89	Muscle contraction	P07951	Tropomyosin beta chain	TPM2	44	1	16.2	1.418	0.744
90	Activation of MAPKK activity	P31946	14-3-3 protein beta/alpha	YWHAB	188	2	24.8	1.201	0.616
91	Protein export from nucleus	P63241	Eukaryotic translation initiation factor 5A-1	EIF5AL1	54	1	7.8	1.105	0.772
92	Keratinization	P35908	Keratin, type II cytoskeletal 2 epidermal	KRT2	1260	21	38.2	0.974	NA
93	Cellular response to calcium ion	P13645	Keratin, type I cytoskeletal 10	KRT10	1903	29	45	0.963	1.831
94	Response to hypoxia	P14618	Pyruvate kinase PKM	PKM	352	10	26.9	0.918	0.811
95	RNA transport	P22626	Heterogeneous nuclear ribonucleoproteins A2/B1	HNRNPA2B1	199	4	18.7	0.881	0.281
96	Histone mRNA metabolic process	P62318	Small nuclear ribonucleoprotein Sm D3	SNRPD3	45	1	11.1	0.931	0.479
97	Positive regulation of protein phosphorylation	P63244	Guanine nucleotide-binding protein subunit beta-2-like 1	GNB2L1	68	1	7.9	0.860	1.189
98	GTP catabolic process	P68104	Elongation factor 1-alpha 1	EEF1A1	543	18	34	0.859	1.596
99	Response to virus	P26641	Elongation factor 1-gamma	EEF1G	195	4	9.6	0.763	1.257
100	Cilium assembly	Q15051	IQ calmodulin-binding motif-containing protein 1	IQCB1	41	1	2	0.759	1.429
101	Cell redox homeostasis	P07237	Protein disulfide-isomerase	P4HB	86	3	7.5	0.710	1.076
102	RNA binding	Q92804	TATA-binding protein-associated factor 2 N	TAF15	56	1	2.9	0.614	0.372

**Figure 3:**
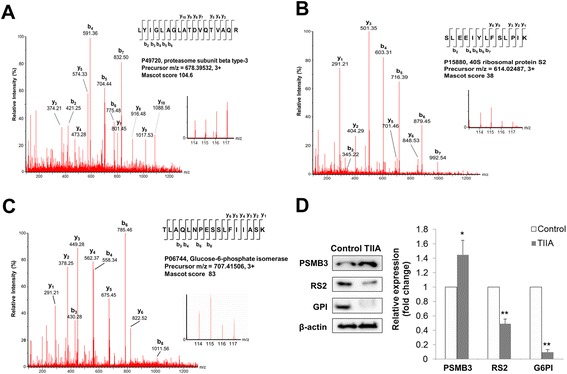
**MS/MS spectra of peptide and protein levels for PSMB3, RS2 and G6PI.** MS/MS spectra of peptides from PSMB3 **(A)**, RS2 **(B)** and G6PI **(C)** are reported along with iTRAQ ion reporter quantification. Ion, m/z 114 and 115 represent peptides collected from control samples. Ion, m/z 116 and 117 represent peptides collected from TIIA-treated samples. **(D)** Protein expression levels of PSMB3, RS2 and G6PI were examined using western blotting analysis, with β-actin as an internal control. All experiments were repeated three times with independent samples.

In order to validate iTRAQ data, we evaluated certain identified protein expression patterns (fold change > 1.5 or < 0.65) by Western blot analysis, including proteasome subunit β type-3 (PSMB3), 40S ribosomal protein S2 (RS2) and glucose-6-phosphate isomerase (G6PI). In our MS/MS spectra, PSMB3 (Figure 3A), RS2 (Figure 3B) and G6PI (Figure 3C) displayed a high intensity across different samples. After western blotting analysis, PSMB3 can be detected to be significantly up-regulated (fold change = 1.45, *p* < 0.05) whereas RS2 (fold change = 0.49, *p* < 0.01) and G6PI (fold change = 0.1, *p* < 0.01) were significantly down-regulated (Figure 3D). As our results, expression patterns of PSMB3, RS2 and G6PI correspond with our iTRAQ data for those proteins, suggesting the consistence between iTRAQ and western blotting data.

### TIIA suppresses glucose metabolism of gastric cancer cells

**Figure 4:**
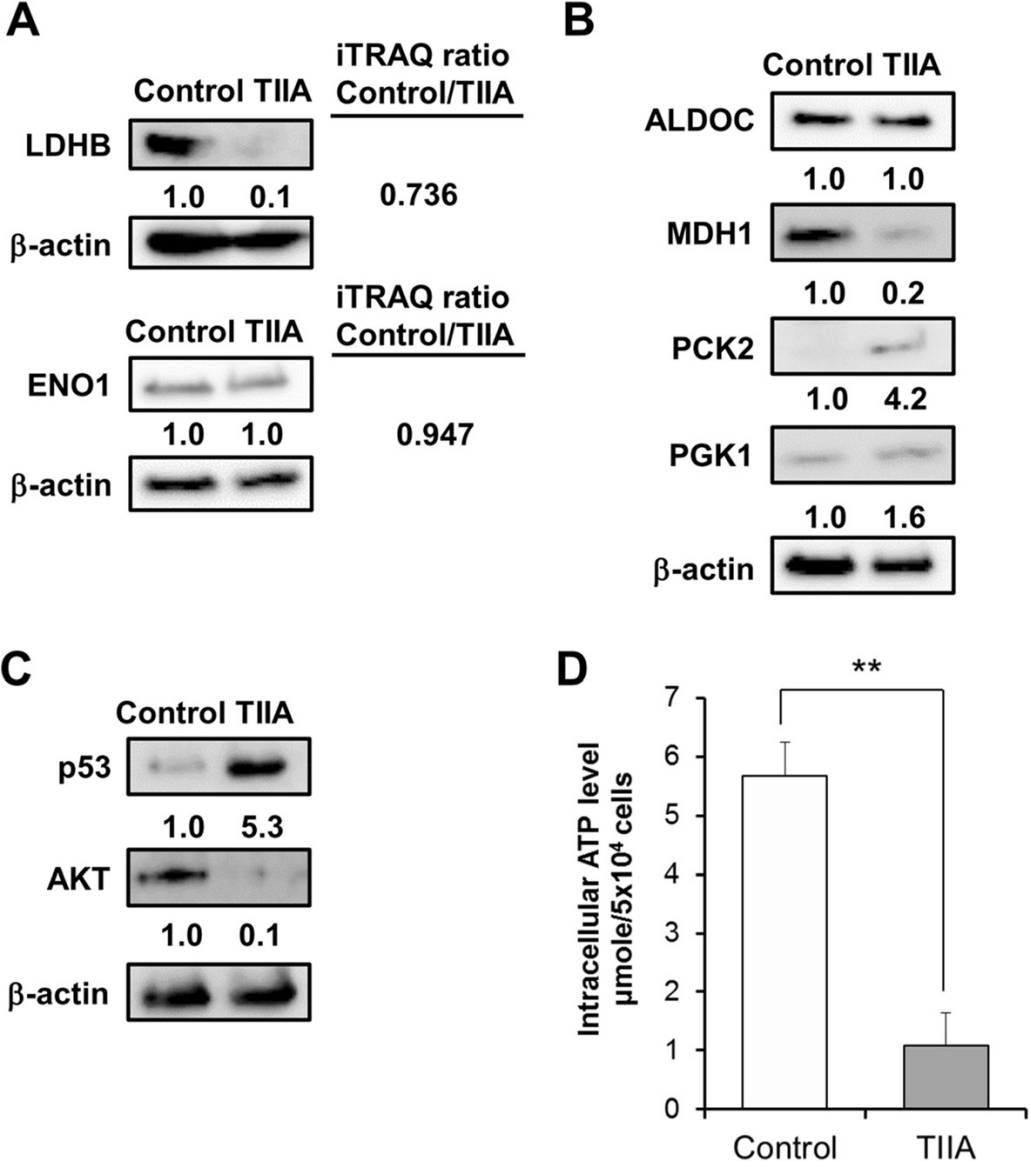
**Treatment with TIIA changes the expression of glucose metabolism-related proteins in AGS cells. (A)** The expression of iTRAQ-identified proteins, LDHB and ENO1 was estimated using western blotting. **(B)** The expression of ALDOC, MDH1, PCK2 and PGK1 in AGS cells treated with TIIA was estimated using western blotting. The levels of **(C)** tumor suppressor gene, p53, and the oncogene AKT, and **(D)** intracellular ATP, were examined in AGS cells treated with TIIA. β-actin was used as an internal control.

To delineate the mechanism of TIIA in gastric cancer cells, we compared our quantitative proteomics and transcriptomics data. We found TIIA regulated not only genes but also proteins involved in glycolysis, the cell cycle, apoptosis, DNA damage and cytoskeleton rearrangement. As shown in Figure 2C, five proteins (PGAM4, ENO1, LDHA, LDHB and G6PI) were identified at both the protein and transcription level. After western blotting analysis, we found that the protein expression of G6PI and LDHB was downregulated by TIIA, and ENO1 was not changed (Figures 3D and 4A). Because G6PI and LDHB are important enzymes in glycolysis and gluconeogenesis, respectively, we further examined if TIIA can regulate other proteins related to glucose metabolism, such as aldolase C (ALDOC), malate dehydrogenase 1 (MDH1), phosphoenolpyruvate carboxykinase 2 (PCK2) and phosphoglycerate kinase 1 (PGK1), which are involved in gluconeogenesis. ALDOC and PCK2 were also identified in our transcriptomics data (Additional file 2). MDH1, which reduces oxaloacetate to malate in the mitochondria, was downregulated by TIIA. PCK2, which converts oxaloacetate to phosphoenolpyruvate, was up-regulated by TIIA (Figure 4B). The transformation of oncogenes, such as p53 and AKT, is also involved in the glucose metabolism switch in cancer cells [18]. We found that p53 increased and AKT decreased in gastric cancer cells following TIIA treatment (Figure 4C). Additionally, TIIA treatment significantly decreased the intracellular ATP levels in AGS cells compared with the control sample (Figure 4D).

### TIIA arrests the cell cycle at the G_2_/M phase transition

**Figure 5:**
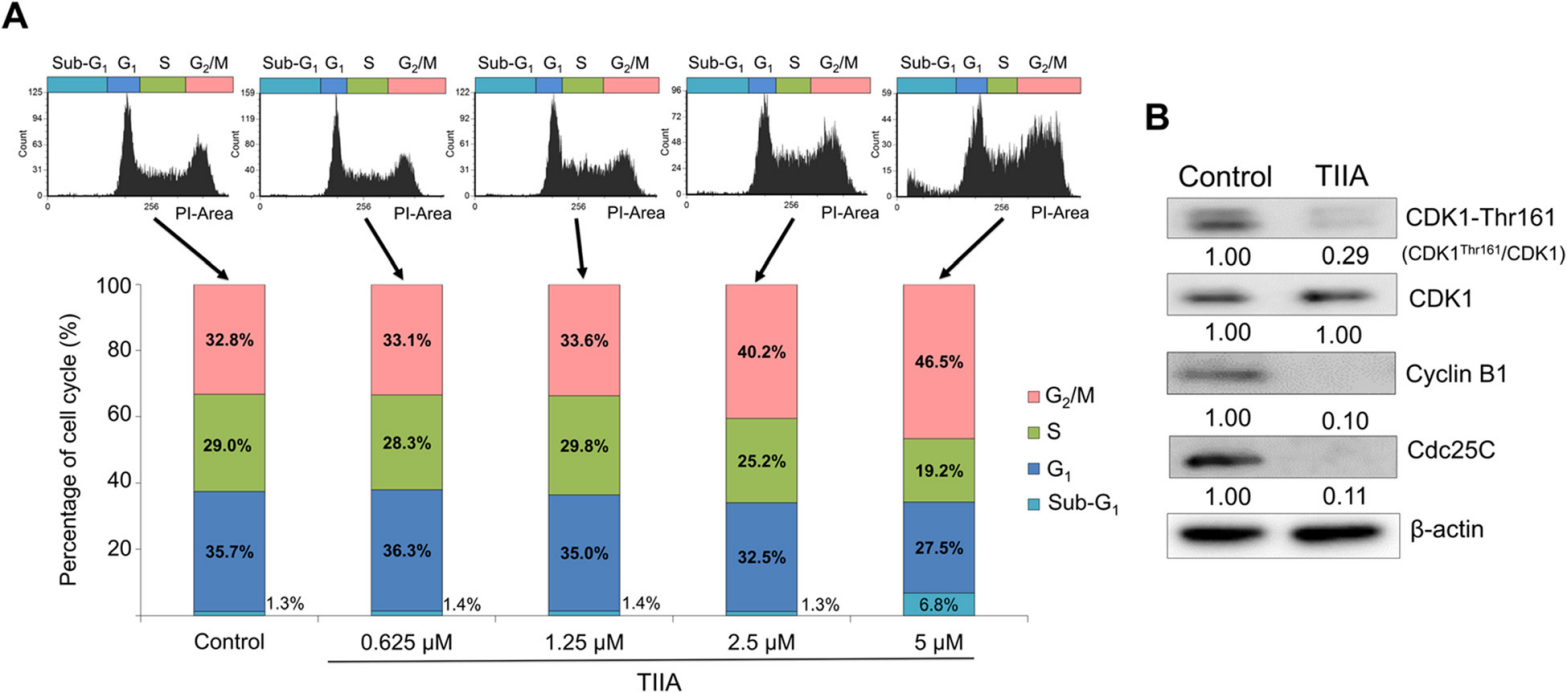
**TIIA induces cell cycle arrest at the G**_**2**_ **/M transition in AGS cells. (A)** Flow cytometric analysis shows the distribution of DNA content in AGS cells after 48 h of TIIA treatment. Cellular DNA was stained by PI and analyzed to quantify the percentage of cells in certain cell cycle phases using FCS Express 4. The percentage of AGS cells in the G_2_/M phase transition increases along with increases in TIIA treatment concentrations, exhibiting a dosage-dependent relationship. The percentage of cells in the sub-G_1_ phase also increases, from 1.3% (control) to 6.8% (5.3 μM TIIA), suggesting the occurrence of apoptosis. **(B)** Protein levels of Phospho-CDK (Thr 161), total CDK, cyclin B1 and Cdc 25C were analyzed using western blotting. β-actin was used as an internal control.

The ability to monitor response to regulation of the cell cycle is an enriched function from our transcriptomics data (Figure 2A and Table 1). We treated AGS cells with different concentrations of TIIA and measured DNA distributions by using flow cytometry to detect the cell cycle distribution of a population of cancer cells. The percentage of AGS cells in the G_2_/M phase increased up to 13.68% above control levels after 5.3 μM TIIA treatment, showing that TIIA induces cell cycle arrest of AGS at G_2_/M in a dosage-dependent manner (Figure 5A).

As indicated in Table 1, CDK1, cyclin B1 and Cdc25C are associated with the cell cycle at the G_2_/M phase, and were identified in our transcriptomics data. CDK1 activation can regulate the progression of the cell cycle from the G_2_ to the M phase, which is dependent on coordination with cyclin B [24,25]. The activation of the CDK1/cyclin-B complex is maintained through phosphorylation of Thr161 and dephosphorylation of Thr14 and Tyr15 in CDK1. Dephosphorylation of Thr14 and Tyr15 in CDK1 is catalyzed by phosphatase Cdc25C, which is considered a rate-limiting step for the G_2_ to M phase transition [24,25]. Previous reports suggest monitoring the alteration of CDK1, cyclin B1, Cdc25C, and phospho-CDK1 (CDK1-Thr161) protein expressions is a useful way to validate the occurrence of cell cycle arrest at the G_2_/M transition [26]. For these reasons, to confirm whether TIIA induces cell cycle arrest at G_2_/M in gastric cancer cells, we treated AGS cells with TIIA at a concentration of 5.3 μM (IC_50_), and then measured protein expression levels using western blotting analysis. Levels of phospho-CDK1 (CDK1-Thr161), cyclin B1, and Cdc25C were all reduced in cells treated with TIIA (Figure 5B). Our results indicate that TIIA treatment induced characteristic cell cycle arrest at G_2_/M in AGS cells by altering cyclin B1 and Cdc25C expression as well as the phosphorylation of CDK1.

### TIIA treatment causes apoptosis and reorganization of actin filaments and microtubules

**Figure 6:**
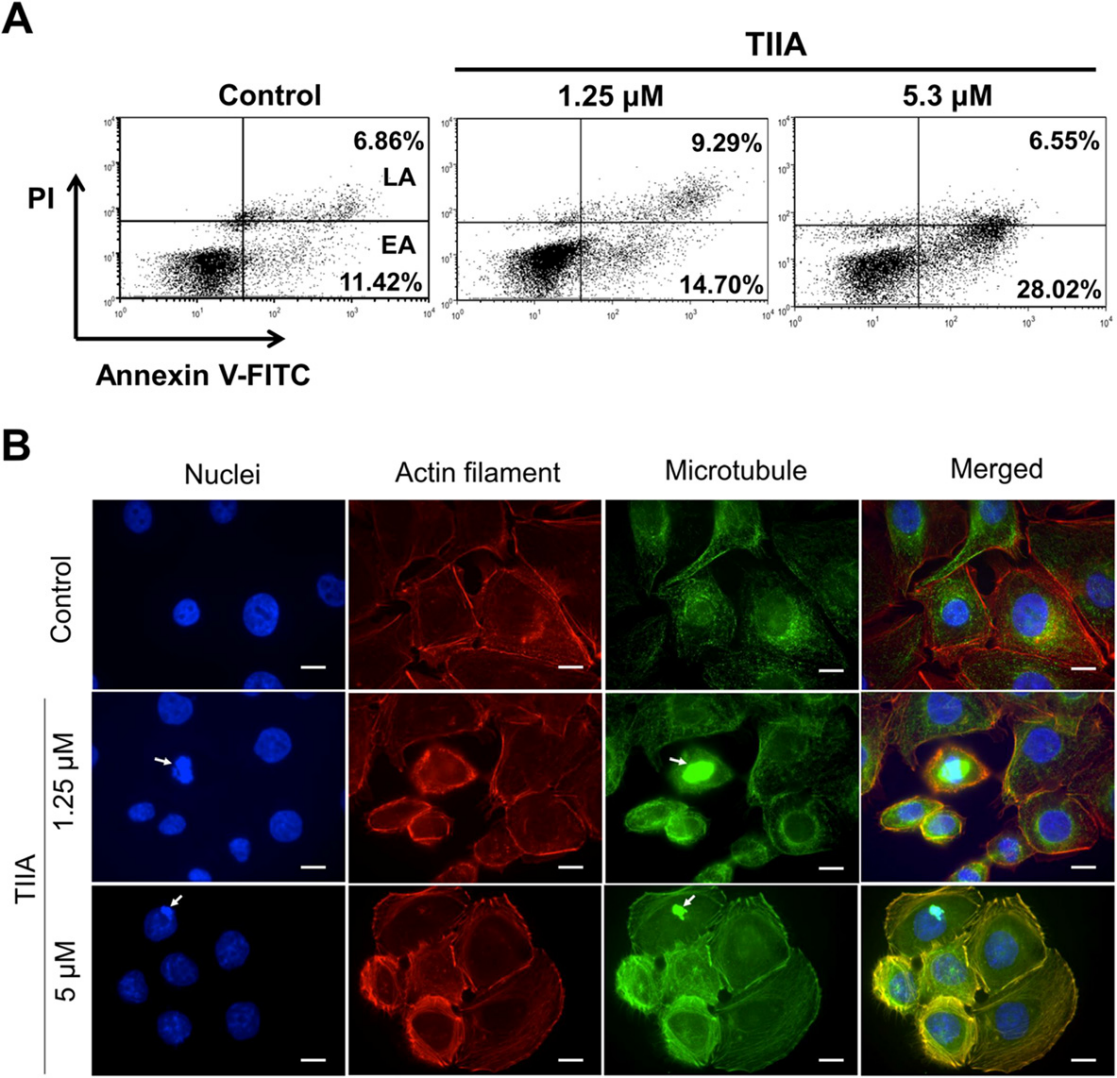
**TIIA induces apoptosis and reorganization of cytoskeleton in AGS cells. (A)** AGS cells were treated with different levels of TIIA (1.25 μM and 5.3 μM). Treated cells were stained with annexin A5 and PI and their apoptotic condition was analyzed by flow cytometry. EA denotes early apoptosis; LA denotes late apoptosis. **(B)** Images of AGS cells were obtained by fluorescence microscopy after TIIA treatment for 48 hr. Nuclei were stained with DAPI (blue), actin filaments were stained with rhodamine-labeled phalloidin (red), and microtubules were stained with mouse anti-α-tubulin antibody and the corresponding FITC-conjugated secondary anti-mouse IgG antibody (green). Arrows indicate nuclear fragmentation sites with condensed chromatin. Microtubules are densely packed at these sites; this condensation is an important step during the apoptotic process [30]. Scale bars represent 10 μm.

Our cell cycle analysis also indicated that levels of cells in the sub-G_1_ phase were increased 5.5% above control levels under 5 μM TIIA treatment conditions (Figure 5A). A significant increase of cells in the sub-G1 phase is widely accepted as a sign of apoptosis induction [27]. As our transcriptomics and proteomics data (Figure 2A and Table 1) show, apoptosis-related genes could be induced by TIIA. We treated AGS cells at different concentrations of TIIA and detected the proportions of cells undergoing apoptosis or necrosis using flow cytometry. Our results show that the proportion of cells undergoing apoptosis (including early- and late-phase apoptosis) significantly increased by 15.3% above control levels under 5.3 μM TIIA treatment conditions, showing that TIIA induced apoptosis of AGS cells in a dosage-dependent manner (Figure 6A).

It is widely understood that reorganization of the cytoskeleton, including actin filaments and microtubules, plays a crucial role in apoptosis [28,29]. Links between this process and TIIA treatment can be seen in our transcriptomics and proteomics data (Figure 2A and Table 1). To detect whether the cytoskeletons of AGS cells undergo reorganization after TIIA exposure, we treated AGS cells with TIIA at different concentrations, then examined the consequent distribution of actin filaments and microtubules using immunofluorescence staining. Many cells were seen to manifest shrinking morphology after TIIA treatment (Figure 6B). Actin filaments under TIIA treatment became more condensed, especially at the cell periphery, and underwent crumbling. On the other hand, microtubules aggregated to become thick bundles, and were distributed along nuclear fragmentation sites with condensed chromatin (Figure 6B). These kinds of cytoskeletal reorganizations, combined with nuclear fragmentation, are all characteristic of apoptosis [2,28-30], showing that TIIA induced cytoskeleton reorganization arising from apoptosis in AGS cells.

### TIIA triggers γ-H2AX nuclear foci in response to DNA double strand breaks

**Figure 7:**
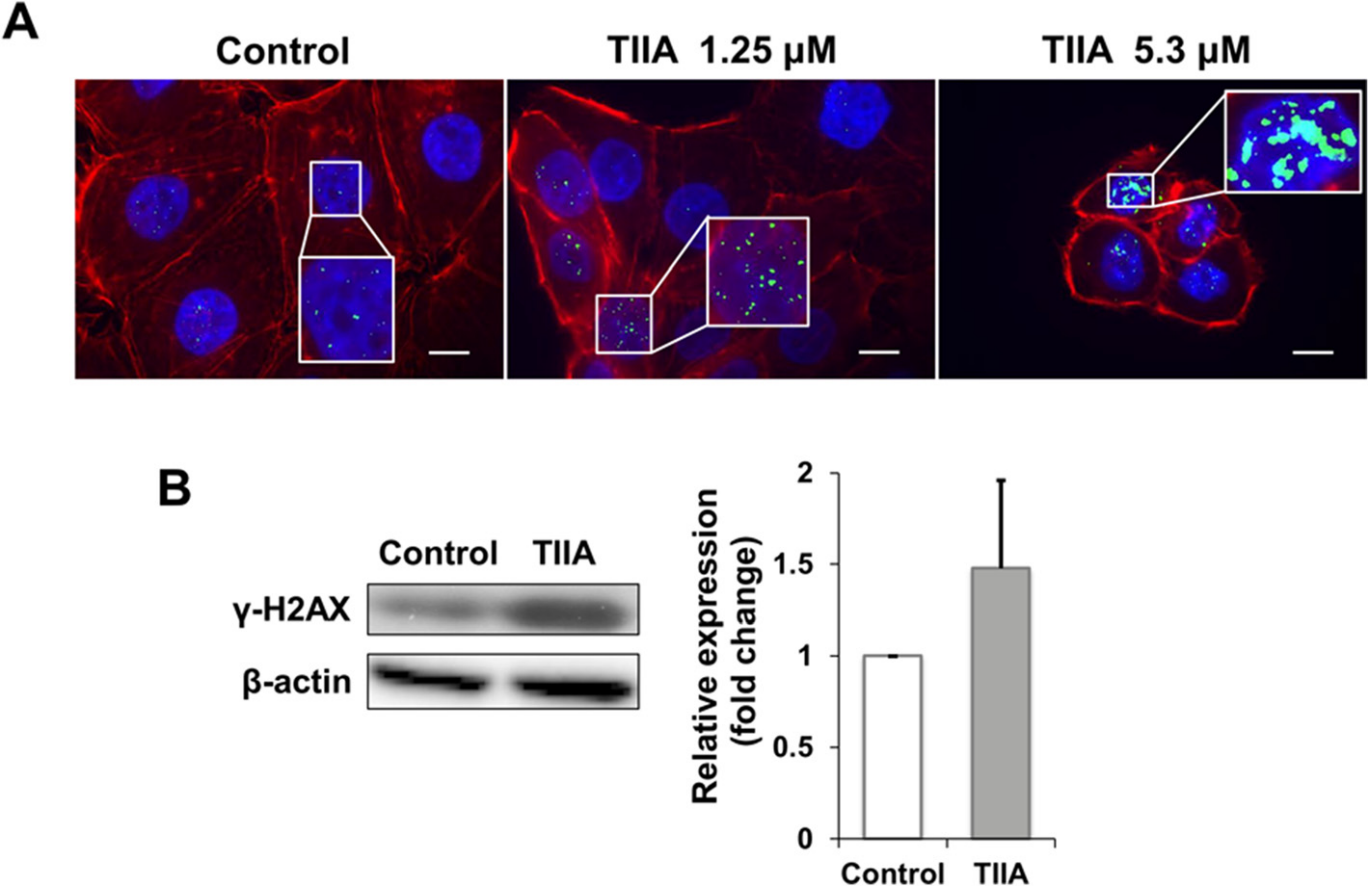
**TIIA triggers DNA double-strand breaks in AGS cells. (A)** Images of AGS cells were obtained by fluorescence microscopy after 48 hr of TIIA treatment. Nuclei were stained with DAPI (blue), actin filaments were stained with rhodamine-labeled phalloidin (red), and γ-H2AX were stained with mouse anti-γ-H2AX antibody and the corresponding FITC-conjugated secondary anti-mouse IgG antibody (green). Each zoomed panel shows representative γ-H2AX distributions in detail. Scale bars represent 10 μm. **(B)** Protein expression of γ-H2AX increases under an IC_50_ dose of TIIA; samples were analyzed by Western blotting with β-actin as internal control. Histogram values for fold change are expressed as mean ± SD from three independent experiments.

Based on our previous results, TIIA could induce DNA damage in gastric cancer cells (Figure 2A and Table 1). DNA damage, including double strand breaks (DSB), often leads to genetic instability; proper cellular responses to DNA damage are crucial for cell function and survival [31,32]. Previous studies have shown that phosphorylation of the histone variant H2AX, producing γ-H2AX at nuclear foci, plays an important role in the DNA damage response triggered by DSB [33,34]. The change in H2AX levels was also apparent in our data (Table 1). To examine whether TIIA triggers DNA damage in gastric cancer cells, we treated AGS cells with different levels of TIIA to examine the subsequent localization of γ-H2AX using immunofluorescence staining. Numerous γ-H2AX foci were localized in the nuclei of TIIA treated cells, while γ-H2AX was only represented in a few foci in control cells (Figure 7A). Increased protein expression of γ-H2AX was also detected in TIIA-treated cells (Figure 7B). These results suggest that TIIA triggers DSB, triggering a DNA damage response in AGS cells.

## Discussion

Botanical herbs have been used for disease treatment and prevention, and as alternative and complementary therapies [35]. For instance, paclitaxel isolated from one kind of botanical herb, *Taxus brevifolia*, has been used as second-line chemotherapy for advanced or recurrent gastric cancer [36]. In this study, we treated gastric cancer with TIIA, which can be used to improve heart function by limiting apoptosis or oxidative damage [37]. Recently, many studies have shown that TIIA exhibits anti-tumor potential [10-16], and that on cancers such as breast cancer, prostate cancer, and leukemia, it can induce the mitochondria-dependent apoptosis pathway [38-40]. In MKN45 gastric cancer cells, TIIA was reported to inhibit cell growth, induce G_2_/M cell cycle arrest and apoptosis [16,41]. However, few of them actually constructed possible pathways or mechanisms in gastric cancer.

Genes for glycolysis are overexpressed in 70% of all human cancers worldwide, including gastric cancer [42]. By comparing our transcriptomics and proteomics data, we are the first to propose that the transformation of glucose metabolism, glycolysis and gluconeogenesis in gastric cancer cells is a major biological process that is regulated by TIIA treatment. An enzyme involved in glycolysis, G6PI, catalyzes the reversible isomerization of glucose-6-phosphate to fructose-6-phosphate, and is downregulated by TIIA [43]. Increased synthesis of G6PI, known as autocrine motility factor (AMF), is considered to be a unique feature of cancer cells, which stimulates cell growth and contributes to cancer metastasis and malignancy [44-47]. AMF can also down-regulate caspase-9 and Apaf-1, making cancer cells more resistant to mitochondria-dependent apoptosis [48]. Previous studies have reported that TIIA can induce mitochondria-dependent apoptosis by regulating caspase-9 and Apaf-1 in several types of cancer [38-40]. In gastric cancer, we also found that TIIA may induce apoptosis (Figure 6A). Based on our results, we suggest that TIIA might induce the occurrence of apoptosis by suppressing G6PI expression, which decreases glucose consumption and inhibits glycolysis in cancer cells.

In gluconeogenesis, expression of LDHB, one of the subunits of lactate dehydrogenase (LDH), which converts lactate to pyruvate, was shown to decrease in AGS cells after TIIA treatment. Pyruvate is the end product of glycolysis and contributes to gluconeogenesis, acetyl-CoA which enters the Krebs (TCA) cycle, lipid synthesis and nonessential amino acid synthesis for proliferative responses in tumor cells [17]. Decreased LDHB expression might enhance negative regulation of the Warburg effect, which mediates pyruvate into lactate and back in tumor cells [49]. In mouse immortalized cell lines, LDHB is critical in the mechanistic target of rapamycin (mTOR) pathway to induce tumor formation [50]. LDHB is also considered as a tumor marker that increases in many cancers because it facilitates tumor growth and cell proliferation [51-53]. Serum LDH is also considered to be predictors for overall survival of advanced nasopharyngeal carcinoma patients [54]. In gastric cancer cells, we suggest that TIIA treatment might block nutrient supply, which reduces cell survival and proliferation by causing lactate dehydrogenase deficiency.

**Figure 8:**
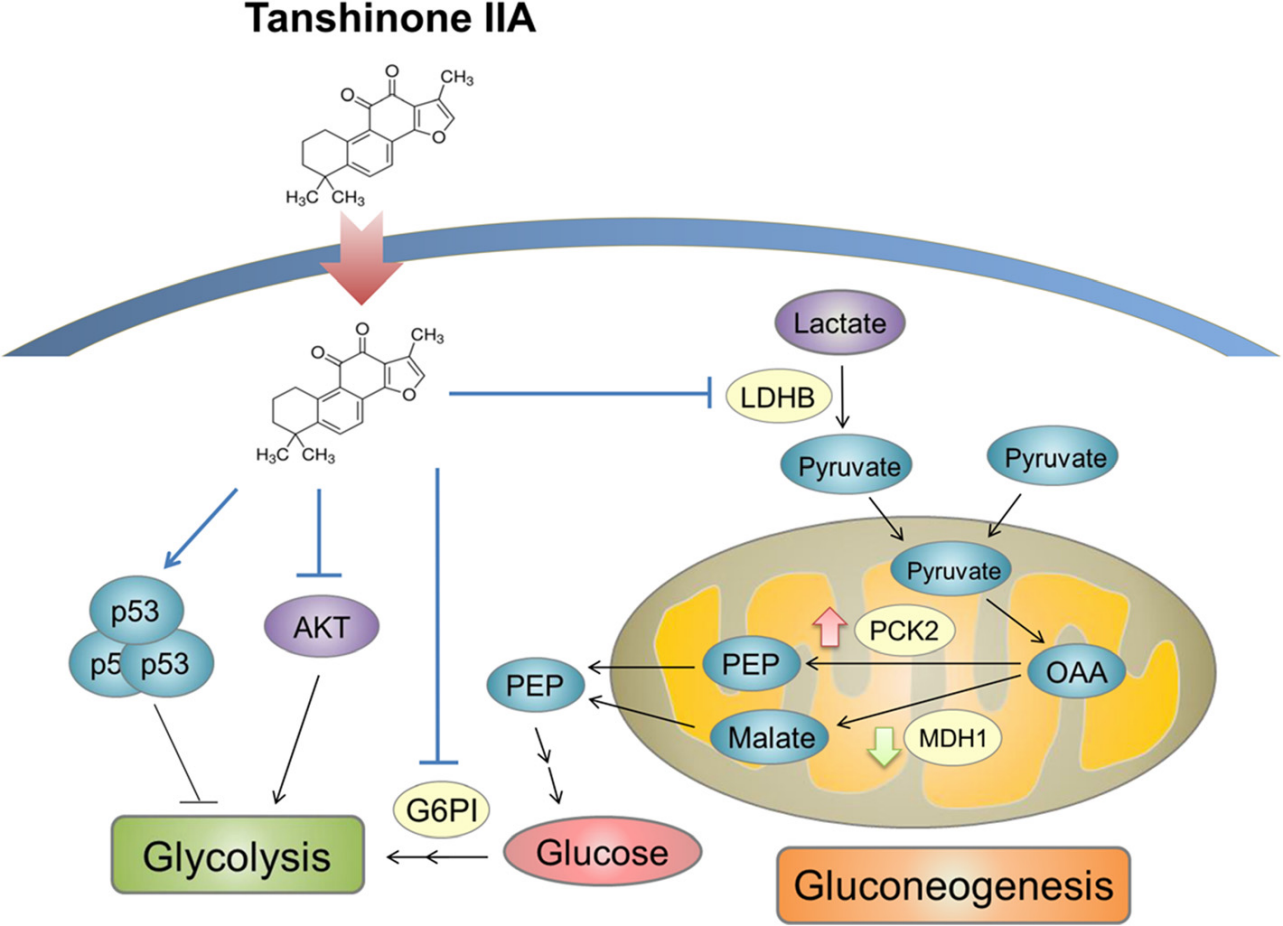
**Schematic representation of TIIA blocking glucose metabolism in gastric cancer cells.** In tumor cells, glucose is consumed to produce ATP, and the glycolytic intermediates are used for biosynthetic pathways. Proto-oncogene, AKT, stimulates glycolysis and the tumor suppression gene, p53, suppresses glucose metabolism via several pathways. After TIIA treatment, intracellular ATP levels and AKT expression decreases, and p53 expression increases. In the second step of glycolysis, glucose-6-phosphate isomerase, acting as an enzyme, was down-regulated by TIIA treatment. We also found that TIIA dysregulates gluconeogenesis by suppressing LDHB and MDH1 expression, and enhancing PCK2 expression. TIIA promotes the activity of these pathways to suppress cancer cell growth.

MDH1 and PCK2 in mitochondria are used to convert pyruvate to phosphoenolpyruvate. In colon cancer, increased MDH levels could enhance glycolysis and lead to cell proliferation and tumorigenesis [55]. Silencing PCK2 in colon cancer cells could reduce susceptibility to preoperative 5-fluorouracil-based radiation therapy [56] and responses to high glucose levels [57]. In this study, we found that TIIA treatment can decrease MDH1 expression and increase PCK2 expression (Figure 4B). Increased PGK1 in gastric cancer is considered as a biomarker of advanced gastric cancer and is followed by increasing intracellular ATP levels [58]. However, TIIA reduced intracellular ATP levels and slightly up-regulated PGK1, which indicates that some functions of TIIA are still unclear. Moreover, the oncogene AKT was down-regulated by TIIA, which can stimulate aerobic glycolysis in cancer cells [59], suggesting that TIIA has the potential to be used as chemotherapy for gastric cancer because of its effectiveness in transforming glucose metabolism (Figure 8).

The glucose metabolic switch is associated with the cell cycle, apoptosis and DNA repair in tumor cells [17]. Our transcriptomics analyses and proteomics data also showed that TIIA can regulate these functions and their related proteins, such as PSMB3 and RS2. Previous studies have shown that the expression of PSMB3 and RS2 is involved the development of cancer [60,61]. PSMB3, which belongs to the proteasome B-type family, was up-regulated by TIIA. Proteasomes are necessary for the DNA damage response, and accumulate at double strand break sites to recruit other repair proteins [62]. TIIA treatment increased the likelihood of the occurrence of DNA damage in gastric cancer cells (Figure 7), which might stimulate cancer cells to increase PSMB3 expression.

Moreover, RS2, as a cancer-related ribosomal protein, was down-regulated by TIIA. Increased RS2 is present when p53 mutates, which occurs in cell cycle progression or apoptosis in response to DNA damage [60]. Elevated expression of p53, cell cycle arrest in the G_2_/M phase, apoptosis and cytoskeleton reorganization were observed in AGS cells following TIIA treatment. DNA damage is one of the major causes of cell cycle arrest in the G_2_/M phase [63]. Cytoskeleton reorganization, including the redistribution of actin and microtubules, is a characteristic of chromatin remodeling during apoptosis [64]. This has also been observed in cancer cells treated with clinical anti-cancer drugs, such as etoposide, doxorubicin and taxol [65].

## Conclusions

We integrate transcriptomics and proteomics data to uncover TIIA-regulated mechanisms in gastric cancer. In glycolysis and gluconeogenesis, TIIA reduces glucose consumption and the production of pyruvate by regulating G6PI, LDHB, MDH1, PCK2 and PGK1 expression. AKT decreases and p53 increases in response to apoptosis and DNA damage in gastric cancer cells following TIIA treatment, while the glucose metabolism switch induced by the transformation of oncogenes is destroyed (Figure 8). We suggest that TIIA treatment could cause cell stress, nutrient deficiency and DNA damage, to induce cycle arrest at the G_2_/M transition, apoptosis and cytoskeleton reorganization, all of which would inhibit cell proliferation. In this study, we provide new insight into the TIIA regulatory mechanism in gastric cancer cells, so that future cancer research can improve therapeutic strategies.

## Methods

### Cell culture

Human stomach adenocarcinoma AGS (CRL-1739; ATCC) cells were grown in 90% RPMI 1640 medium (Biological Industries, Beit Haemek, Israel) supplemented with 10% fetal bovine serum (Biological Industries). Cells were cultured at 37°C in an incubator with controlled humidified atmosphere containing 5% CO_2_. Cells were disaggregated for subculturing with trypsin plus ethylenediaminetetraacetic acid (EDTA), then spun down by centrifugation at 1200 rpm for 5 min, after which the pellet was re-suspended in culture medium.

### TIIA preparation

Tanshinone IIA powder (T4952; Sigma-Aldrich) was dissolved in dimethyl sulfoxide (DMSO) (BioShop), which was diluted to applicable concentrations for treatment.

### Growth curves and IC_50_ measurement

Growth curves of AGS cells were recorded by an RTCA DP**®** system (xCELLigence™; Roche Applied Science & ACEA Biosciences). Cells were treated with TIIA or 0.1% DMSO as control for 72 hr after 24 hr of seeding (5,000 cells/well in 96-well E-plates). TIIA treatment concentrations were 0.1 μM, 1 μM, 5.3 μM, and 10 μM; 0.1% DMSO dissolved in culture medium was used as control. Each condition was performed in triplicate. The IC_50_ of TIIA at 48 hr was calculated with RTCA software 1.2 (xCELLigence™) by using AGS cell growth curves under TIIA treatments of 0.1 μM, 1 μM, 5.3 μM, and 10 μM.

### Colony formation assay

AGS cells were treated with TIIA or 0.1% DMSO control medium for 5 days after 24 hr of seeding (2,500 cells/well in 6-well plates). TIIA treatment concentrations were 0.53 μM, 1.06 μM, and 5.3 μM; 0.1% DMSO dissolved in culture medium was used as control. After treatment, cells were fixed with methanol for 10 min, stained with 200 μg/mL crystal violet (Sigma-Aldrich) for 10 min, and then colonies were counted using AlphaView SA 3.4.0 (ProteinSimple). Each condition was performed in triplicate.

### RNA sequencing and data analysis

Total RNA was extracted from AGS cells treated with DMSO (control) or TIIA, using TRIzol reagent (Invitrogen, USA) according to the manufacturer’s instructions. The quantity and quality of RNA were checked using an Agilent 2100 Bioanalyzer (Agilent Technologies, Santa Clara, CA), and were found to have an RNA Integrity Number (RIN) value of more than 9. Poly(A) mRNA was isolated with oligo(dT)-bound magnetic beads and incubated with fragmentation buffer to form short RNA fragments. Reverse transcriptase and random hexamer primers were used to synthesize the first-strand cDNA, and DNA polymerase I and RNaseH were used to synthesize the second-strand cDNA. Double stranded cDNA was end-repaired with T4 DNA polymerase, Klenow fragments, and T4 Polynucleotide Kinase, after which a single “A” base was added, and the entire sequence was ligated to Illumina sequencing adapters (Illumina, San Diego, CA). PCR was performed to amplify the fragments. Using Illumina HiSeqTM 2000 (Illumina), the cDNA library was sequenced on a flow cell after validation on an Agilent 2100 Bioanalyzer and ABI StepOnePlus Real-Time PCR System.

After the base calling, the reads with adaptor sequences and low quality scores were removed. The reads with low quality scores are defined as the reads with greater than 10% of unknown bases (N) or greater than 50% of low quality bases which quality values are less than 5. The reads with high quality scores were mapped to the human reference genome hg19 assembly using SOAPaligner in SOAP2 with 2 mismatch allowance [66] and annotated based on the GENCODE [67]. Read counts for individual GENCODE genes were subsequently determined using HTSeq-count (http://www-huber.embl.de/users/anders/HTSeq), by considering only uniquely mapped reads. Expression of each individual gene was quantified by using the Reads Per Kilobase per Million mapped reads (RPKM) method. The differentially expressed genes were identified by Poisson distribution model [68]. The sequences reported in this study have been deposited in the Sequence Read Archive database with accession number SRP049450.

### Functional enrichment analysis

We used MetaCore version 6.18 (GeneGo, St. Joseph, MI, USA) to perform a gene function analysis and to enrich the functional networks of identified genes collected from RNA-seq. There were 16,110 of 16,603 identified genes eligible for network enrichment via GO Processes and Metabolic Networks analysis. For analysis of differentially expressed genes (|log_2_(fold–change)| > 1), 2,717 of 2,761 DEGs were eligible for GO Processes enrichment analysis, and displayed classified genes (Table 1). All enrichment analysis was tested using the *p*-value threshold *p* < 0.0001 for the data inputs.

### iTRAQ labeling and LC-MS/MS analysis

AGS cells were treated with 5.3 μM (IC_50_) TIIA or 0.1% DMSO control medium for 48 hr after 24 hr of seeding (8 × 10^4^ cells/well in 6-well plates). Cells were harvested with trypsin/EDTA and then total proteins were extracted by using lysis buffer (1% SDS (Bioman), 50 mM Tris–HCl (pH 6.8), 10% glycerol), and 1× protease inhibitor (Bioman) and sonication. Proteins were processed through reduction, alkylation, and gel-assisted trypsin digestion overnight to yield peptides, which were later extracted from the gels as previous methods [69]. Equal amounts of peptides from control and TIIA-treated samples were labeled by different iTRAQ reagents (AB SCIEX; control samples labeled by 114 or 115; TIIA-treated samples labeled by 116 or 117) and incubated at room temperature for 1 hr. Peptides were combined together and dried with a centrifugal evaporator (CVE-2000; EYELA). After iTRAQ-labeling, samples were desalted and analyzed by using a LC-ESI-Q-TOF mass spectrometer (Waters SYNAPT® G2 HDMS; Waters Corp.). Samples were injected into a 180 mm × 2 cm capillary trap column and separated by a 75 mm × 25 cm nanoACQUITY UPLC™ 1.7 mm Ethylene Bridged Hybrid C18 column using a nanoACQUITY Ultra Performance LC™ System (Waters Corp.). The mass spectrometer (MS) was operated in electrospray ionization sensitivity mode, and calibrated with a synthetic human [Glu1]-Fibrinopeptide B solution (1 pmol/ml; Sigma-Aldrich) delivered through a NanoLockSpray™ source, which was used for accurate mass measurements. Data was acquired in the data directed analysis (DDA) mode, which included one full MS scan (m/z 350–1700, 1 s) and three sequential MS/MS scans (m/z 100–1990. 1.5 s for each scan) on the three most intense ions present in the full scan mass spectrum.

### Protein identification

The peak list resulting from MS/MS spectra was generated by Mascot Distiller v2.3.2 (Matrix Science, London, United Kingdom). Data files were searched against the sequence database (containing 536,789 sequences entries) of the Swiss-Prot human database, using Mascot search engine v2.3.02 (Matrix Science, London, United Kingdom). Both the precursor peptide and fragment ion tolerances were set to ±0.1 Da. The search parameter settings were as follows: allowances for two missed cleavages from trypsin digestion and variable modifications of deamidation (NQ), oxidation (M), iTRAQ (N terminal), iTRAQ (K), and methylthio (C). The peptide charge was set to M_r_, the instrument was set to ESI-QUAD-TOF, and the decoy database was searched. Mascot search results were filtered using “Significance threshold” set at *p* < 0.05 and “Ions score or expect cut-off” set at 0.05. To evaluate the false discovery rate (FDR), we compared a decoy database search against a randomized decoy database created by Mascot using identical search parameters and validation criteria. FDR was calculated as *D/R* × 100%, where *D* and *R* are the number of matches above identity threshold using the decoy and real databases, respectively. The mass spectrometry proteomics data have been uploaded to the ProteomeXchange Consortium [70] via the PRIDE partner repository with the data set identifier PXD000998 and DOI 10.6019/PXD000998.

For protein quantitation, signature ions (m/z = 114, 115, 116 and 117) and peptides were detected and analyzed using Multi-Q software (v1.6.5.4) [71]. Peptides that satisfied the following four criteria were subjected to further analysis. Firstly, the peptide is labeled with iTRAQ tags; secondly, the peptide has an ion score higher than the Mascot identity score (*p* < 0.05); thirdly, the peptide is nondegenerate (unique); fourthly, the iTRAQ signature ion peak intensity (ion count) of the peptide is within the dynamic range (the peak intensity of each iTRAQ signature ion must be > 0, and the average of the peak intensities of all iTRAQ signature ions must be ≥ 30). Before quantitation of the expression of each protein, the peak intensity of the iTRAQ signature ion was normalized, as “Method 1” of our previous study [72]. To determine the expression ratio of identified proteins in AGS cells from both the control and the TIIA treatment, the normalized peptide iTRAQ signal of each identified protein was summarized, to calculate protein ratios (TIIA treatment/control).

### Western blot analysis

AGS cells were treated with 5.3 μM (IC_50_) TIIA or 0.1% DMSO control medium for 48 hr after 24 hr of seeding (8 × 10^4^ cells/well in 6-well plates). Cells were harvested with trypsin/EDTA and total proteins were extracted. Then, proteins from control and TIIA-treated samples were separated in 12% SDS-PAGE gels, and transferred onto 0.45 μm PVDF membranes (Millipore) in a Trans-Blot® SD Semi-Dry Transfer Cell (Bio-Rad) for 50 min at 400 mA. The membrane was blocked for 1 hr at room temperature in 5% non-fat milk powder/PBS-T (1× PBS, 0.1% Tween 20 (Sigma-Aldrich)) and incubated overnight at 4°C with blocking buffer containing rabbit monoclonal antibodies to human RS2 (GeneTex; 1:1,000), PSMB3 (GeneTex; 1:1,000), phospho-CDK1 (Santa Cruz; 1:100), CDK1 (Santa Cruz; 1:100), Cyclin B1 (GeneTax; 1:500), Cdc25C (GeneTex; 1:1,000), G6PI (GeneTex; 1:1000), ENO1 (GeneTex; 1:2000), MDH1 (GeneTex; 1:500), PGK1 (GeneTex; 1:500), ALDOC (GeneTex; 1:250), PCK2 (GeneTex; 1:1000), LDH-B (GeneTex; 1:100), p53 (Santa Cruz; 1:500) or AKT (Santa Cruz; 1:1000). The membrane was washed with PBS-T, incubated 1 hr with 5% non-fat milk powder/PBS-T containing anti-rabbit IgG antibodies (1:10,000) (Sigma-Aldrich) or anti-mouse IgG antibodies (Sigma-Aldrich, 1:10,000), washed and imaged with enhanced chemiluminescence (PerkinElmer). The membrane image was then analyzed by an AutoChemi Image System (UVP) or exposed to Fuji medical X-ray film, followed by quantification with AlphaView SA 3.4.0 (ProteinSimple).

### Intracellular ATP generation assay

Cells were seeded onto 6-well plates at 8 × 10^4^ cells/well, and then incubated for 24 h. For the control, 0.1% DMSO was added to the medium, and for the treatment, 5.3 μM TIIA was added. After 48 h of drug exposure, the medium was removed, and then cells were washed twice with PBS. The levels of intracellular ATP were determined using a bioluminescent somatic cell assay kit (Sigma-Aldrich), according to the manufacturer’s instructions, and normalized to protein concentrations. Luminescence was detected using a FlexStation III (Molecular Devices). The ATP content of each sample was calculated as the average of the relative light readings and based on the ATP standard curve.

### Flow cytometry

For cell cycle analysis, AGS cells were treated with TIIA or DMSO as control for 48 hr. TIIA treatment concentrations were 0.625 μM, 1.25 μM, 2.5 μM, and 5.3 μM; 0.1% DMSO dissolved in culture medium was used as control. After treatment, cells were harvested with trypsin/EDTA, fixed with 70% ethanol, then spun down, after which ethanol was removed. Then each sample was mixed with RNase A (100 μg/mL), incubated at 37°C for 1 hr, and stained with propidium iodide (PI) (Santa Cruz Biotechnology, Inc.) at a concentration of 100 μg/mL in the dark at room temperature for 15 min. For apoptosis analysis, AGS cells were treated with TIIA or DMSO control medium for 48 hr after 24 hr of seeding (3.5 × 10^5^ cells in 10-cm plates). TIIA treatment concentrations were 1.25 μM, and 5.3 μM; 0.1% DMSO dissolved in culture medium was used as control. After treatment, cells were harvested with trypsin/EDTA, suspended, and counted. Then each sample was adjusted to a concentration of 10^6^ cells/tube and stained with Annexin V-FITC (Santa Cruz Biotechnology, Inc.) and PI (Santa Cruz Biotechnology, Inc.) dissolved in binding buffer (Santa Cruz Biotechnology, Inc.) in the dark at room temperature for 15 min. Both cell cycle distribution and apoptotic cells proportion were then analyzed with a BD FACSCanto II flow cytometer (BD Biosciences) and FCS Express 4 (BD Biosciences).

### Immunofluorescence staining

AGS cells were treated with TIIA or DMSO control medium for 48 hr after 24 hr of seeding (4 × 10^4^ cells in 6-well plates). TIIA treatment concentrations were 1.25 μM and 5.3 μM, and 0.1% DMSO dissolved in culture medium was used as control. Cells were washed, fixed with 4% paraformaldehyde (Sigma-Aldrich) in PBS for 20 min at 37°C, then permeabilized with 0.25% Triton X-100 (Sigma-Aldrich) in PBS for 10 min at room temperature. Then cells were incubated with 1% BSA (Bioshop) in PBS as blocking buffer for 30 min at room temperature, and labeled with mouse monoclonal antibodies to human α-tubulin (Millipore; 1:500) or γ-H2AX (Abcam; 1:500) dissolved in blocking buffer at 4°C overnight. After being washed with PBS three times, cells were labeled with anti-mouse FITC-IgG (Millipore; 1:100) and TRITC-conjugated Phalloidin (Millipore; 1:2000) dissolved in blocking buffer for 1 hr in the dark at room temperature. Then cells were washed with PBS three times and mounted with ProLong® Gold reagent with DAPI (Invitrogen). Images were acquired by using a fluorescence microscope with Leica HCX FL PLAN 1006/1.25 oil objective, a SPOT camera (Diagnostic Instruments), and were analyzed with SPOT Advanced software (Diagnostic Instruments).

### Statistical analysis

Data were expressed as mean ± standard deviation (SD) and analyzed using two-tailed Student’s *t*-tests. In the cell proliferation assays, data were analyzed using the Wilcoxon Signed-Rank test. A *P*-value of less than 0.05 was taken to indicate statistical significance.

## Additional files

Additional file 1:
**The scatter plot represents gene expression levels (mRNA-RPKM) in the control and TIIA treatment samples.** Colored dots represent DEGs (FDR ≤ 0.001 and |log_2_(fold–change)| ≥ 1). Additional file 2:
**List of RNA-seq identified genes and their expression ratios (TIIA/Control).**
Additional file 3:
**The top 50 enrichment functions of RNA-seq-identified DEGs analyzed by MetaCore software.**
Additional file 4:
**Detailed information on iTRAQ identified proteins and peptide ratios (TIIA/Control).**
Additional file 5:
**High reproducibility of iTRAQ-based proteomics analysis. (A)** Control sample peptides were equally divided into two parts, C1 and C2, and labeled with iTRAQ 114 and 115 separately. The distribution plot of iTRAQ ion intensity in control samples (711 peptides identified) shows a high correlation (R^2^ = 0.975) between the two labels. **(B)** Treatment (IC_50_ dose of TIIA for 48 hr) sample peptides were equally divided into two parts, T1 and T2, and labeled with iTRAQ 116 and 117 separately. The distribution plot of iTRAQ ion intensity in treatment samples (711 peptides identified) shows a high correlation (R^2^ = 0.984) between the two labels.
