# Supplementary material for: Integrating transcriptomics and proteomics to show that tanshinone IIA suppresses cell growth by blocking glucose metabolism in gastric cancer cells
Source: BMC Genomics. 2015 Feb 5;16(1):41. doi: 10.1186/s12864-015-1230-0 (PMC4328042; doi:10.1186/s12864-015-1230-0)
Supplement: Additional file 1: — The scatter plot represents gene expression levels (mRNA-RPKM) in the control and TIIA treatment samples. Colored dots represent DEGs (FDR ≤ 0.001 and |log2(fold–change)| ≥ 1). [file 12864_2015_1230_MOESM1_ESM.doc]

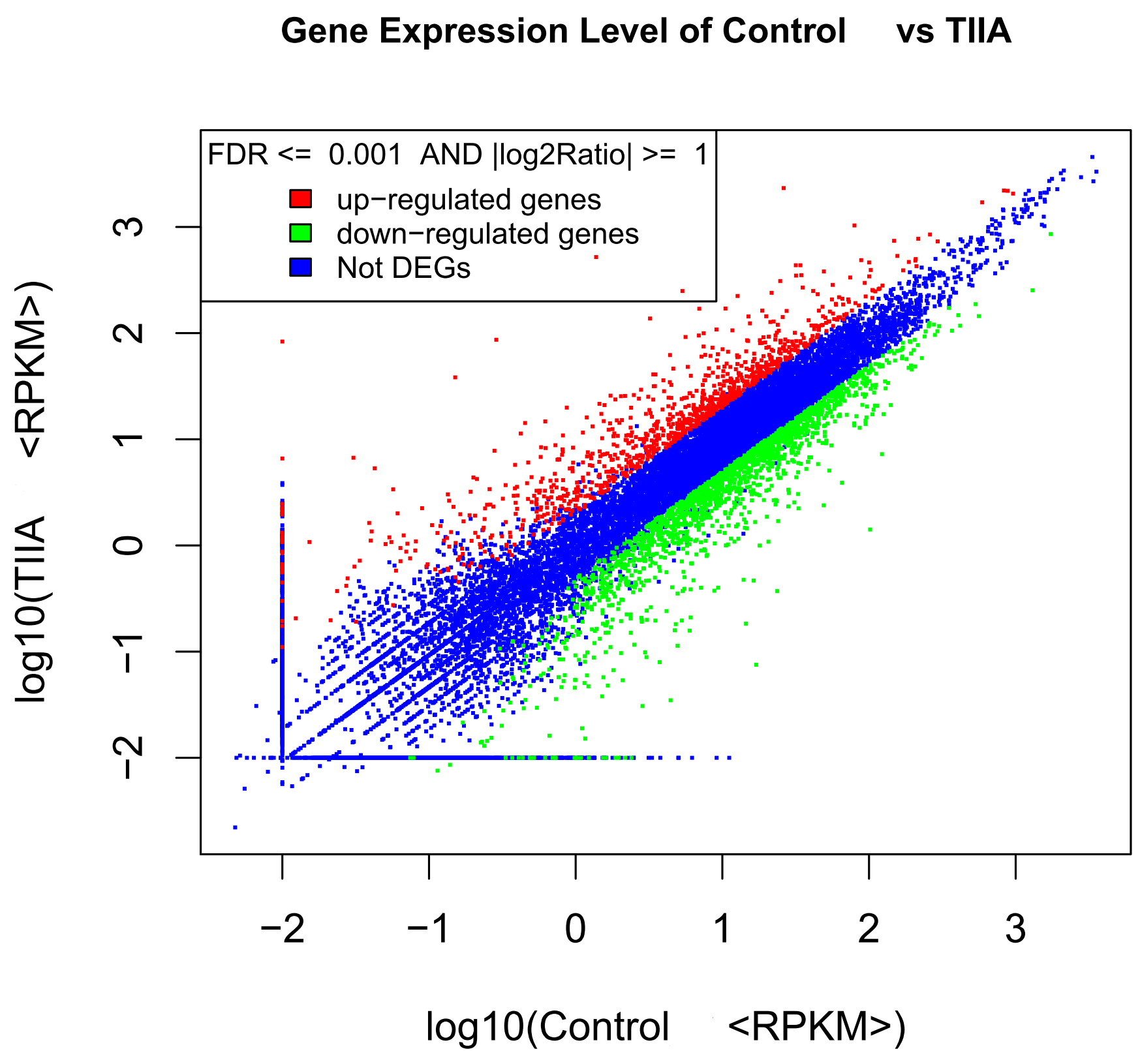


**Additional file 1.** The scatter plot represents gene expression levels (mRNA-RPKM) in the control and TIIA treatment samples. Colored dots represent DEGs (FDR ≤ 0.001 and |log2(fold–change)| ≥ 1).
