# Supplementary material for: Integrating transcriptomics and proteomics to show that tanshinone IIA suppresses cell growth by blocking glucose metabolism in gastric cancer cells
Source: BMC Genomics. 2015 Feb 5;16(1):41. doi: 10.1186/s12864-015-1230-0 (PMC4328042; doi:10.1186/s12864-015-1230-0)
Supplement: Additional file 5: — High reproducibility of iTRAQ-based proteomics analysis. (A) Control sample peptides were equally divided into two parts, C1 and C2, and labeled with iTRAQ 114 and 115 separately. The distribution plot of iTRAQ ion intensity in control samples (711 peptides identified) shows a high correlation (R2 = 0.975) between the two labels. (B) Treatment (IC50 dose of TIIA for 48 hr) sample peptides were equally divided into two parts, T1 and T2, and labeled with iTRAQ 116 and 117 separately. The distribution plot of iTRAQ ion intensity in treatment samples (711 peptides identified) shows a high correlation (R2 = 0.984) between the two labels. [file 12864_2015_1230_MOESM5_ESM.doc]

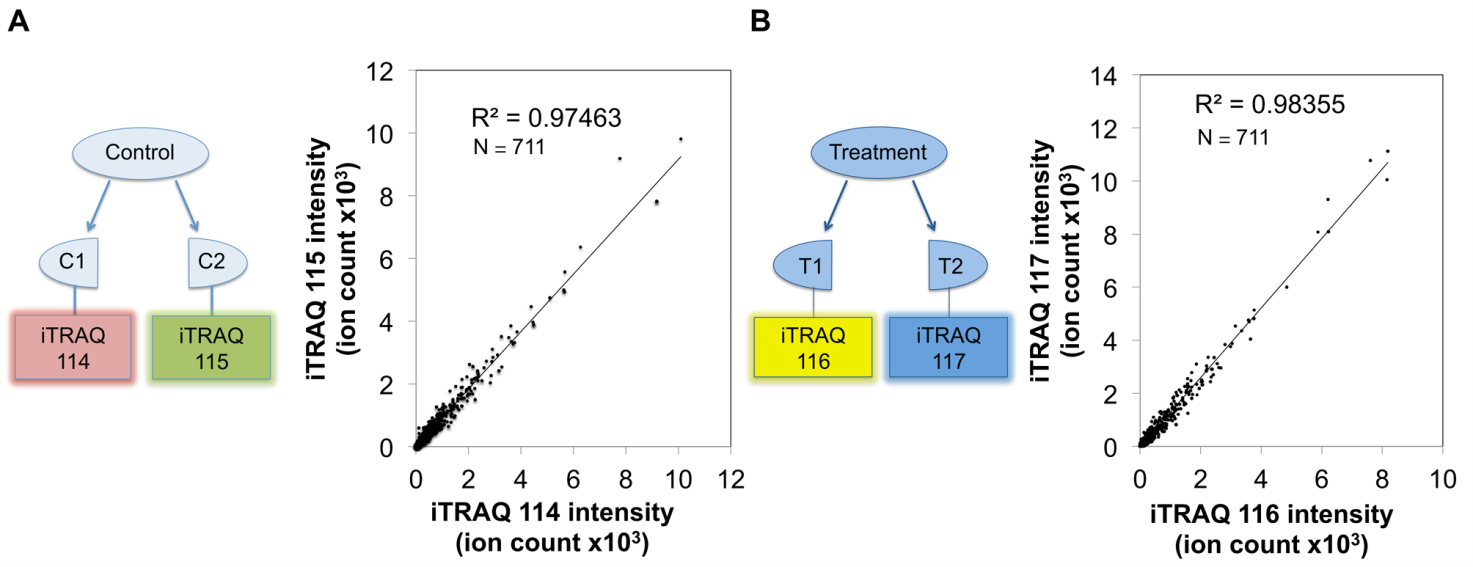


**Additional file 5.** High reproducibility of iTRAQ-based proteomics analysis. (A) Control sample peptides were equally divided into two parts, C1 and C2, and labeled with iTRAQ 114 and 115 separately. The distribution plot of iTRAQ ion intensity in control samples (711 peptides identified) shows a high correlation (R^2^ = 0.975) between the two labels. (B) Treatment (IC_50_ dose of TIIA for 48 hr) sample peptides were equally divided into two parts, T1 and T2, and labeled with iTRAQ 116 and 117 separately. The distribution plot of iTRAQ ion intensity in treatment samples (711 peptides identified) shows a high correlation (R^2^ = 0.984) between the two labels.
